# Impact of a charge nurse development program on nurses’ confidence: a quasi-experimental study in a multinational tertiary hospital in the United Arab Emirates

**DOI:** 10.3389/fmed.2026.1764344

**Published:** 2026-07-16

**Authors:** Bahaa Aldein Naim Shtaih, Zahra Mohamed, Mohammad Mahmoud Khader, Princiy Thomas

**Affiliations:** Sheikh Shakhbout Medical City, Abu Dhabi, United Arab Emirates

**Keywords:** charge nurse, confidence, leadership development, multicultural workforce, nursing education, quasi-experimental, self-efficacy, United Arab Emirates

## Abstract

**Background:**

Charge nurses are pivotal frontline leaders in acute care, yet many assume the role without formal preparation, particularly in multicultural health systems. Confidence in leadership, clinical judgment, and communication is central to safe and effective charge nurse practice, but evidence from Gulf-region tertiary hospitals remains limited.

**Aim:**

To evaluate the effectiveness of a structured Charge Nurse Development Program (CNDP) in improving nurses’ confidence in leadership, clinical, and professional responsibilities within a large multinational tertiary hospital in the United Arab Emirates.

**Methods:**

A quantitative quasi-experimental pre–post design was conducted with 103 nominated registered nurses working in inpatient and critical care units. The three-phase CNDP combined self-directed e-learning, a didactic workshop with simulation and case-based activities, and three supervised charge nurse shadowing shifts, guided by Bandura’s Self-Efficacy Theory, adult learning principles, and experiential learning. Confidence was measured before and 1 week after the program using the 21-item Confidence in Managing Challenging Situations Scale (CMCS). Data were analyzed using paired-samples *t*-tests, Welch’s ANOVA, and multiple linear regression; effect sizes and internal consistency (Cronbach’s α) were reported.

**Results:**

Confidence improved significantly across all 21 items and both subscales. Leadership & Ethical Practice increased from 27.48 ± 5.31 to 32.95 ± 4.05, and Clinical & Communication Confidence from 36.26 ± 7.01 to 43.56 ± 5.43; total confidence rose from 63.74 ± 11.74 to 76.51 ± 9.26 (all *p* < 0.001, d ≈ 0.82–0.87). Cronbach’s α for the Confidence Scale was 0.963. Baseline differences by credential favored charge nurses, but post-intervention scores no longer differed significantly. No meaningful differences were found across nationality, age, qualification, years of experience, or unit, and regression analyses showed no consistent demographic predictors of change.

**Conclusion:**

The CNDP was associated with educationally meaningful improvements in nurses’ self-reported leadership and clinical confidence in a highly multicultural workforce. These findings suggest that structured, theory-informed charge nurse development may support perceived readiness for frontline leadership responsibilities. Further controlled and longitudinal studies using objective behavioral, clinical, and organizational outcomes are needed to determine whether confidence gains translate into sustained practice change.

## 1 Introduction

Charge nurses hold a pivotal leadership role in acute care environments, serving as the operational and clinical coordinators responsible for managing patient flow, staffing allocations, real-time decision-making, and escalation processes. Their actions influence interdisciplinary communication, teamwork, nurse well-being, and overall patient safety (2, 3). Effective performance in this role requires rapid situational interpretation, prioritization, conflict resolution, and the emotional intelligence needed to support staff during routine and emergent conditions. A substantial body of evidence links competent charge nurse leadership with improved team functioning, reduced burnout, and enhanced patient outcomes shaping the organizational culture (4, 5).

Global nursing shortages have intensified the need for strong frontline leadership. Recent reports highlight critical associations between leadership practices, staff engagement, retention, and patient safety, underscoring the importance of developing leadership capacity at the unit level (6–8). With the World Health Organization projecting a deficit of 4.5 million nurses by 2030, strengthening charge nurse leadership is essential to promoting workforce stability and health-system resilience (9).

Despite the complexity of the charge nurse role, many nurses assume these responsibilities without formal preparation a phenomenon described as “accidental leadership,” whereby role selection is based on availability or seniority rather than competency or readiness (10). This unstructured transition is often associated with reduced confidence, role ambiguity, and difficulty navigating unit-level challenges (11). In multinational workforces such as those in the UAE, differing cultural norms in communication, delegation, and authority can further complicate role expectations and leadership consistency (12, 13). These contextual factors highlight the need for structured, competency-based leadership development tailored to diverse clinical environments.

Although charge nurse development programs have demonstrated positive outcomes in various settings including improvements in leadership readiness, communication, and clinical decision-making few have been evaluated in the Gulf region, and none have integrated a theory-based, multi-phase design with a validated confidence measure in a large, multicultural tertiary hospital (1, 14). This gap underscores the need for context-specific evidence to guide leadership development within diverse healthcare systems.

This study therefore evaluated the effectiveness of a structured three-phase Charge Nurse Development Program (CNDP) in enhancing nurses’ confidence in a multinational tertiary hospital in the United Arab Emirates. Guided by Bandura’s Self-Efficacy Theory, the program incorporated mastery experiences, vicarious learning, social persuasion, and emotional regulation strategies mechanisms known to strengthen confidence and leadership capability. Using a quasi-experimental pre–post design and validated confidence measure, this study contributes new evidence regarding leadership capacity-building in complex, multicultural healthcare environments.

## 2 Literature review

### 2.1 Charge nurse role complexity and leadership demands

Literature consistently identifies charge nurses as operational leaders essential to patient flow, escalation, and decision-making during each shift (2). Their performance influences nurse well-being, team cohesion, and clinical quality indicators (4). Yet, many assume the role without adequate preparation, reporting gaps in confidence, conflict management, prioritization, and delegation (11). Emerging evidence confirms that the charge nurse role is far more complex than traditionally perceived, requiring nurses to function as clinical-administrative leaders who integrate leadership, communication, problem-solving, and deep organizational awareness into real-time decision-making (15). Studies consistently show that charge nurses must balance significant managerial and supervisory responsibilities under high operational pressure (16–18), yet these competencies are rarely taught during initial preparation, leaving many nurses underprepared for frontline leadership (15, 19–21).

### 2.2 Confidence and self-efficacy in nursing leadership

Confidence is a core determinant of self-efficacy and a predictor of leadership behavior, resilience, communication ability, and problem-solving in clinical practice. High self-efficacy has been associated with improved nurse leadership performance, higher engagement, and better coping under stress (22). Leadership self-efficacy has also been linked to improved coping under workload demands, enhanced teamwork, and stronger perceptions of work engagement and quality of care (23).

Conversely, a study by Aboalrob et al. (24) demonstrates that nurses with higher professional self-concept show significantly stronger clinical decision-making capabilities. This implies that nurses with lower self-concept or confidence are more vulnerable to decision hesitation and suboptimal judgment, reinforcing evidence that low confidence contributes to decision avoidance and weaker leadership behavior. These findings underscore the importance of structured educational interventions that build mastery experiences, provide role modeling, and enhance emotional regulation key mechanisms through which self-efficacy develops in Bandura’s framework.

### 2.3 Effectiveness of charge nurse development programs

Evidence from quasi-experimental and pilot studies consistently demonstrates that structured charge nurse development programs can substantially enhance frontline leadership capability. Dols et al. (25), for example, showed that a structured, evidence-based charge nurse education curriculum significantly improved participants’ leadership readiness, communication efficiency, and capacity to coordinate unit-level operations. Their findings underscore the value of explicit leadership role socialization and structured skill-building in mitigating the ambiguity that often characterizes unprepared transitions. Similarly, (26, 27) claimed improvements in leadership style, resilience, and self-assurance when structured development program and stated that charge nurses are still not adequately prepared to lead. Their work reinforces the psychological impact of supportive leadership training, particularly in high-pressure clinical environments where emotional regulation and rapid decision-making are essential. Additionally, Kramer and Davies (10) demonstrated increased competence, clarity of responsibilities, and self-confidence among nurses transitioning into charge roles after structured orientation. Their findings suggest that early socialization into leadership expectations can prevent the uncertainty and role conflict commonly associated with unstructured transitions.

In the Gulf region, Hamed et al. (14) conducted an ADDIE-based Charge Nurse Training Program in a Saudi NICU and found significant gains in knowledge, communication, and perceived leadership readiness, illustrating the feasibility of structured charge-nurse training in Middle Eastern contexts. This reinforces the applicability of structured leadership interventions across culturally diverse healthcare systems.

Systematic reviews echo these empirical findings. Cummings et al. (28) concluded that leadership-focused educational interventions not only strengthen frontline nursing leadership skills but also positively influence engagement, retention, and communication across clinical teams. Despite this progress, most charge nurse development studies have been conducted in North American or single culture hospitals and empirical evidence from highly diverse, multinational Gulf-region tertiary hospitals remains limited. Aiken et al. (29) further emphasized that well-prepared frontline leaders particularly charge nurses play a pivotal role in shaping practice environments, influencing patient outcomes, and enhancing team cohesion. Collectively, these studies provide strong evidence that structured charge nurse development programs are essential for building the leadership capacity required in modern healthcare systems.

### 2.4 Theoretical framework

This study is underpinned by Bandura’s Self-Efficacy Theory, which provides a robust psychological explanation for how individuals develop confidence in performing complex tasks. Bandura (22) conceptualized self-efficacy as the belief in one’s ability to organize and execute the actions required to manage prospective situations. These beliefs are shaped through four interrelated mechanisms: mastery experiences, vicarious learning, social persuasion, and the regulation of emotional and physiological states. Together, these processes influence how individuals interpret challenges, exert effort, and persist in the face of difficulty.

Within the context of charge nurse leadership, self-efficacy theory offers a compelling lens through which to understand the development of confidence in clinical decision-making, communication, delegation, and crisis management. Mastery experiences, considered the strongest source of self-efficacy, occur when nurses successfully navigate leadership tasks such as handling emergencies, resolving conflict, or coordinating unit operations. Vicarious learning, gained through observing peers or mentors perform similar responsibilities, further strengthens leadership self-belief by providing models of effective practice. Social persuasion, including supportive feedback and coaching, contributes by reinforcing individuals’ perceptions of their capability. Finally, emotional and physiological regulation such as managing stress during high-acuity events shape confidence by influencing how leaders interpret their internal states when facing demanding situations.

Although Bandura’s theory provided the primary framework for understanding confidence development, Kolb (30) Experiential Learning Cycle and Knowles (31) Adult Learning Theory strengthened the educational design of the CNDP. Kolb’s framework was relevant because charge nurse development requires movement between experience, reflection, conceptual understanding, and application in practice. Knowles’ adult learning theory was also appropriate because the participants were experienced nurses who brought prior clinical knowledge, required learning that was relevant to their role, and benefited from self-directed and practice-based activities. Together, these frameworks were complementary: Bandura explained how confidence and self-efficacy could be strengthened, Kolb guided the experiential and reflective structure of learning, and Knowles supported the use of learner-centered strategies suited to adult professional nurses.

This theoretical alignment informed the structure and content of The Charge Nurse Development Program (CNDP). Simulation-based activities and case scenarios provided structured mastery opportunities, while facilitated debriefings and peer observation supported vicarious learning. Faculty coaching and reflective dialogue offered social persuasion, and structured discussions on stress management and emotional awareness promoted effective self-regulation. Through these mechanisms, the CNDP aligns closely with Bandura’s framework, creating conditions that would theoretically enhance charge nurses’ confidence across leadership, clinical, and interpersonal domains. These complementary theories provide a conceptual foundation for the CNDP by supporting experiential learning, self-directed professional development, and the enhancement of participants’ self-reported confidence in leadership-related activities rather than demonstrating objective leadership competence or behavioral change.

### 2.5 Research aim and questions

#### 2.5.1 Aim

To evaluate the effectiveness of a structured Charge Nurse Development Program (CNDP) in improving nurses’ confidence in performing leadership, clinical, and professional responsibilities.

#### 2.5.2 Research questions

1. Does the CNDP significantly improve nurses’ confidence scores from pre- to post-intervention?
2. Are there differences in pre- and post-confidence levels across demographic characteristics (gender, age, marital status, nationality, qualification, years of experience, unit, credential)?
3. Does the use of the validated Confidence Scale Walsh et al. (1) demonstrate acceptable reliability in this UAE context?
4. What demographic or professional factors, if any, influence the change in confidence after the CNDP?

## 3 Materials and methods

### 3.1 Study design

This study employed a quantitative quasi-experimental pre–post design to determine whether participation in the Charge Nurse Development Program (CNDP) resulted in significant improvements in nurses’ confidence. This design is widely used in nursing leadership and educational intervention research where randomization is not feasible (25, 26). Although quasi-experimental designs carry inherent threats to internal validity (history, maturation, testing effects, and selection bias), using the same participants for both measurement points strengthens internal comparison by enabling within-subject change analysis. Pre-intervention data were collected immediately before the program began, and post-intervention data were collected 1 week after completing all CNDP phases. The pre–post structure allowed for direct measurement of changes in nurses’ confidence associated with the educational intervention.

### 3.2 Setting

The study took place in a 700 bed tertiary teaching hospital in Abu Dhabi, United Arab Emirates. The hospital provides specialty services across intensive care, emergency, medical and surgical units, perioperative care, maternity, pediatrics’, and ambulatory clinics. The nursing workforce is highly multinational, representing more than 60 nationalities. This multicultural composition, combined with the complexity of care and the institution’s academic mission, makes the setting ideal for evaluating leadership capacity building interventions.

### 3.3 Participants and sampling strategy

A purposive, nomination-based sampling strategy was used. Unit Managers identified registered nurses who demonstrated leadership potential or were expected to assume charge responsibilities. These nominations were based on clinical performance, interest, and professional readiness, making the sampling a purposive nomination-based approach, followed by voluntary participation. A total of 154 nurses were nominated, 121 consented to participate, however 103 completed the different phases of the program and completed both pre- and post-tests. Reasons for non-completion among the 18 participants who consented but did not complete all program phases and both pre- and post-assessments were not systematically collected. Although nomination-based sampling enhances relevance for leadership development programs, it introduces selection bias, which is acknowledged as a limitation of this design. All participants were registered nurses currently employed in direct patient care within the hospital. Nurses were eligible if they, (a) worked in clinical areas where charge responsibilities are required or anticipated, (b) were nominated by their unit managers based on their readiness and willingness to attend the program, and (c) agreed to complete the program and both pre- and post-intervention surveys.

Exclusion criteria included Unit Managers, administrative or office-based nursing staff who were not patient-facing, and fresh graduate nurses, as they were either already in formal leadership roles or were not expected to assume charge nurse responsibilities during the study period.

### 3.4 Sample size calculation

Sample size adequacy was determined a priori using G*Power software (Version 3.1.9.4) for a paired-samples *t*-test (32). A medium effect size (Cohen’s *d* = 0.50), α = 0.05, and power = 0.80 indicated a minimum of 100 participants. The achieved sample (*N* = 103) met this requirement, yielding post-hoc power of approximately 0.82.

### 3.5 Intervention: Charge Nurse Development Program (CNDP)

The Charge Nurse Development Program (CNDP) was designed as a multi-modal educational intervention based on principles from Adult Learning Theory (Knowles), Kolb’s Experiential Learning Cycle, and Bandura’s Self-Efficacy Theory. These frameworks emphasize self-directed learning, experiential practice, reflective development, and mastery-based competency acquisition–making them well suited for charge nurse leadership preparation.

The program was developed and delivered by a different functional specialty of nursing and nursing education team consisting of the Nursing Education Manager, Clinical Resource Nurses, Nurse Supervisors, the Nursing Quality Manager, Nursing Excellence officer and the Case Management Manager. The curriculum was implemented across three structured phases, integrating e-learning, interactive workshops, and supervised clinical immersion to scaffold learning from foundational knowledge to applied leadership practice. Drawing on quality reports, different unit safety incidents and concerns, case management data, and clinical resource nurses’ direct observation of unit practices, the team collaboratively identified recurrent gaps in charge nurses’ and team leaders’ leadership, communication, and clinical coordination skills. All taskforce members held at least a Master’s degree in nursing or quality and relevant specialty certifications, as well as extensive experience in teaching and clinical experience. This expert group translated observed practice gaps into targeted learning objectives, scenarios, and workshop content, ensuring that the program was both contextually relevant and pedagogically robust as provided in Table 1.

**TABLE 1:** Structure of the charge nurse development program.

Phase	Description	Key content areas	Educational strategies/theoretical link
Phase 1: pre-course online modules	Self-paced foundational learning prior to workshops	Leadership & management; interprofessional communication; change management; time management; patient communication	Knowles’ Adult Learning Theory: self-directed, relevance-based learning
Phase 2: didactic course and workshops	Instructor-led training with role plays, discussions, reflection	Charge nurse roles; team coordination; escalation; NDNQI and RCA; discharge processes; conflict management; resilience and stress	Kolb’s Experiential Cycle: abstract conceptualization; vicarious learning; reflective observation
Phase 3: supervised shadowing (3 shifts)	Real-world practice under supervision of nurse manager	Unit management; patient flow; staffing adjustments; escalation; safety oversight	Bandura’s Self-Efficacy Theory: mastery experience, feedback, social persuasion

Phase 1: Pre-course Online Learning (4–6 h)

Participants completed self-directed modules on leadership principles, interprofessional communication, change management, time management, and effective communication with patients. This phase operationalized Knowles’ adult learning principles by promoting autonomy, relevance, and flexibility.

Phase 2: Didactic Course and Interactive Workshops (8 h)

The in-person workshop included charge nurse roles, team motivation, escalation pathways, quality and safety (NDNQI, RCA), conflict management, resilience building, and patient flow processes. Case discussions, structured reflection, and role plays supported vicarious learning and critical thinking, consistent with Bandura’s and Kolb’s models.

Phase 3: Supervised Charge Nurse Shadowing (3 clinical shifts)

Participants completed three supervised charge nurse shifts using a standardized checklist. Direct coaching and feedback from nurse managers enabled real-time mastery, reflective learning, and strengthened self-efficacy through hands-on leadership practice.

Attendance, participation and completion of each phase were monitored throughout all phases to ensure intervention fidelity.

The CNDP evaluation was informed by Kirkpatrick’s four-level evaluation model. This study assessed Level 1 (Reaction) through participant satisfaction measures and Level 2 (Learning) through changes in self-reported leadership confidence. Evaluation of Level 3 (Behavior) and Level 4 (Results) was beyond the scope of the present study.

### 3.6 Data collection procedures

Prior to data collection, ethical approval was secured from the Research Ethics Committee of the Institutional Review Board (IRB) (SSMCREC-562) of the participating tertiary hospital. The program and data collection were conducted between January and May 2025. The questionnaire was created using Google Forms to ensure efficient and centralized data collection while maintaining participant anonymity and confidentiality. The survey link, accompanied by an invitation letter and an electronic informed consent statement, was distributed via an institutional email list to all eligible nurses. By proceeding with the survey, participants provided their informed consent. Upon completion of the data collection period, responses were screened for completeness and accuracy. Responses were stored on a secure, password-protected platform accessible only to the research team.

Data collection began with a demographic questionnaire, which gathered information on age, gender, years of experience, unit assignment, and nationality. This was followed by the pre-intervention Confidence Scale developed by Walsh et al. (1), which was distributed via institutional email before the program. The post intervention Confidence Scale was sent after a week from completion of the program. These two time points aligned with the program’s aim to measure learning and self-efficacy gains across the full intervention. All 103 participants completed both assessments, enabling complete paired-samples analysis.

To assess immediate knowledge gain from the structured workshops, a short knowledge test was administered:

- Pre-test: At the start of the in-person didactic session (Phase 2)
- Post-test: Directly after completing the same session

This test measured short-term acquisition of leadership and operational concepts taught during Phase 2. Scores were used descriptively to evaluate Kirkpatrick Level 2 learning outcomes.

At the conclusion of Phase 2 workshops, participants completed a structured program evaluation survey assessing: (a) Overall satisfaction, (b) Perceived relevance and applicability, (c) Teaching effectiveness, (d) Perceived improvement in readiness and (e) Recommendations for program enhancement. These data were summarized using descriptive statistics to evaluate training reaction outcomes (Kirkpatrick Level 1).

### 3.7 Instrumentation: the Confidence Scale

The Confidence in Managing Challenging Situations Scale (CMCS) developed by Walsh et al. (1) was used to measure charge nurses’ self-reported confidence before and after the intervention. The instrument consists of 21 items divided into two structured parts, each scored on a five-point Likert scale ranging from 0 = No confidence to 4 = High confidence.

Part One includes 9 items assessing confidence in professional, ethical, and relational competencies (e.g., understanding legislation on vulnerable adults, advocating for patients, recognizing distress, and acting as a change agent). Part Two contains 12 items measuring confidence in managing complex clinical scenarios (e.g., unexpected emergencies, cardiac arrest, managing deteriorating patients, breaking bad news, challenging poor practice, and communicating within the multidisciplinary team). Scores from both parts are summed to produce a Total Confidence Score (range 0–84), with interpretation guidance provided in the instrument (Low: 0–29, Confident: 29–60, High: 61–84).

For this study, the full 21-item scale was used. Internal consistency reliability in this sample was excellent, with a Cronbach’s alpha of α = 0.963 for the total scale, consistent with the validation study. Although Likert scales are ordinal, the sum of more than 20 items approximates continuous data, justifying the use of parametric tests (33).

### 3.8 Statistical analysis

Data were analyzed using IBM SPSS Statistics version 29. Descriptive statistics summarized demographic characteristics and baseline confidence scores. Normality of pre–post difference scores was assessed using Shapiro–Wilk tests and Q–Q plots; although several items showed deviations from normality, parametric tests were retained given their robustness in samples of this size and the approximated continuity of summed Likert-scale data. Outliers were screened using a ± 3 SD criterion, with no extreme values identified. Homogeneity of variance across demographic groups was tested using Levene’s test, and Welch’s ANOVA was applied when this assumption was not met. Independence of observations was confirmed.

Pre–post changes in confidence were evaluated using paired-samples *t*-tests, with effect sizes reported as Cohen’s d and corresponding 95% confidence intervals. Bonferroni adjustments were applied to item-level analyses to reduce Type I error inflation. Between-group differences were examined using independent-samples *t*-tests for binary demographic variables (gender, marital status) and Welch’s ANOVA for multi-category variables (age, nationality group, qualification, years of experience, unit, and credential). Effect sizes for these comparisons were reported using omega-squared (ω^2^). Statistical significance was set at *p* < 0.05.

## 4 Results

### 4.1 Demographic characteristics of participants

A total of 103 nurses completed both the pre- and post-intervention surveys. The majority were female (83.5%), married (89.3%), and between 34 and 44 years of age (51.5%). Most participants were Expatriate Asian nurses (66.0%), followed by Expatriate Arabs (22.3%) and smaller groups of Emirati, African, and Western nurses. In terms of professional characteristics, three-quarters had more than 10 years of experience (74.8%), and most held a Bachelor of Science in Nursing (78.6%). Participants predominantly worked as Staff Nurses (56.3%), followed by Team Leaders (29.1%) and Charge Nurses (14.6%). The largest clinical areas represented were Medical–Surgical Inpatients (31.1%) and Critical Care (22.3%), with smaller proportions from the Operating Room, Outpatient Department, Pediatrics, Emergency, Health Support Services, and Maternity. Some nationality subgroups had small sample sizes (*n* < 5), so any inferential analyses involving these categories were interpreted with caution (see Table 2).

**TABLE 2:** Demographic characteristics of participants (*N* = 103).

Variable	Category	*n* (%)
Gender	Female	86 (83.5)
	Male	17 (16.5)
Age group	Under 25 years	2 (1.9)
	25–33 years	31 (30.1)
	34–44 years	53 (51.5)
	45–54 years	17 (16.5)
Marital status	Married	92 (89.3)
	Single	11 (10.7)
Nationality	Expatriate Asian	68 (66.0)
	Expatriate Arab	23 (22.3)
	Emirati (UAE national)	7 (6.8)
	Expatriate African	3 (2.9)
	Expatriate Western	2 (1.9)
Years of experience	1–5 years	8 (7.8)
	6–10 years	18 (17.5)
	More than 10 years	77 (74.8)
Nursing qualification	Diploma	6 (5.8)
	BSN	81 (78.6)
	MSc	16 (15.5)
Credential	Staff Nurse	58 (56.3)
	Team Leader	30 (29.1)
	Charge Nurse	15 (14.6)
Unit/working area	Medical surgical inpatients	32 (31.1)
	Critical care unit	23 (22.3)
	Operating room	12 (11.7)
	Outpatient department	11 (10.7)
	Pediatric medical-surgical	7 (6.8)
	Health support services	7 (6.8)
	Emergency department	6 (5.8)
	Maternity and labor	5 (4.9)

Percentages are calculated based on the total sample size (*N* = 103). BSN, Bachelor of Science in Nursing; MSc, Master of Science. Oncology participants were grouped under Medical–Surgical Inpatients for descriptive reporting because of the very small subgroup size.

As this was a single-group pre–post design, no baseline statistical comparisons between demographic groups were required.

The sample reflects the demographic diversity typical of tertiary hospitals in the UAE, with strong representation from multinational nursing groups. A majority of participants possessed substantial clinical experience, indicating that the program reached a seasoned workforce with existing leadership exposure. The distribution across clinical units ensures that the findings capture the leadership needs of nurses working in varied practice environments.

### 4.2 Assumption checking

Assumptions underlying the parametric analyses were examined prior to testing. For the paired-samples *t*-tests, Shapiro–Wilk tests conducted on the pre–post difference scores for all 21 confidence items indicated significant deviations from normality (*p* < 0.001; however, visual inspection of Q–Q plots showed approximately linear distributions, supporting the suitability of paired-sample analyses. Given the large sample size (*n* = 103) and the established robustness of *t*-tests to non-normality in moderate to large samples, the tests were considered appropriate. No extreme outliers were identified, with no values exceeding ±3 standard deviations from the mean. For group comparisons, Levene’s tests indicated unequal variances across several demographic categories; therefore, Welch’s ANOVA was used, which adjusts for heterogeneity of variances. These diagnostics supported the suitability of the chosen parametric analyses.

### 4.3 Pre–post changes in confidence

Paired-samples *t*-tests demonstrated statistically significant improvements across all items and both subscales of the Confidence Scale (*n* = 103). Subscale 1 (Leadership & Ethical Practice) increased from 27.48 ± 5.31 to 32.95 ± 4.05 (Δ = 5.48, *t*(102) = 8.78, *p* < 0.001, *d* = 0.87), while Subscale 2 (Clinical & Communication Confidence) increased from 36.26 ± 7.01 to 43.56 ± 5.43 (Δ = 7.30, *t*(102) = 8.30, *p* < 0.001, *d* = 0.82). Overall confidence rose from 63.74 ± 11.74 to 76.51 ± 9.26 (Δ = 12.78, *t*(102) = 8.83, *p* < 0.001, *d* = 0.87).

Visual inspection of confidence intervals showed no overlap between pre- and post-test means for either subscale or the total score, supporting the magnitude and consistency of the improvement. Large effect sizes (*d* = 0.82–0.87) indicate practically meaningful gains in leadership and clinical confidence following completion of the Charge Nurse Development Program. Item-level results are provided in Table 3.

**TABLE 3:** Item-level pre–post differences in Confidence Scale.

Subscale/item	Pre-test M ± SD	Post-test M ± SD	Δ (Post–Pre)	*t*(102)	*P*	D
Subscale 1: Leadership & Ethical Practice						
Understand legislation (vulnerable adults)	2.82 ± 0.71	3.61 ± 0.51	0.80	9.57	<0.001	0.94
Support/promote health, rights and dignity	3.16 ± 0.68	3.71 ± 0.48	0.55	7.12	<0.001	0.70
Advocate and manage ethical challenges	3.03 ± 0.75	3.65 ± 0.50	0.62	7.11	<0.001	0.70
Work in partnership with service users/families	3.17 ± 0.64	3.66 ± 0.52	0.50	5.91	<0.001	0.58
Build therapeutic relationships	3.20 ± 0.69	3.71 ± 0.48	0.50	6.48	<0.001	0.64
Enable patient decisions and choices	3.08 ± 0.75	3.64 ± 0.52	0.56	6.56	<0.001	0.65
Recognize distress and respond effectively	3.10 ± 0.71	3.67 ± 0.51	0.57	6.96	<0.001	0.69
Act as change agent and provide leadership	2.97 ± 0.73	3.64 ± 0.50	0.67	8.16	<0.001	0.80
Be self-aware regarding values and assumptions	2.96 ± 0.73	3.66 ± 0.50	0.70	8.58	<0.001	0.85
Subscale 1 total (9 items)	27.48 ± 5.31	32.95 ± 4.05	5.48	8.78	<0.001	0.87
Subscale 2: Clinical & Communication Confidence						
Unexpected clinical emergency	3.09 ± 0.69	3.66 ± 0.52	0.57	6.96	<0.001	0.69
Managing group of patients (mentor absent)	2.99 ± 0.77	3.61 ± 0.53	0.62	6.70	<0.001	0.66
Caring for a dying patient	2.97 ± 0.91	3.57 ± 0.59	0.60	5.97	<0.001	0.59
Cardiac arrest	3.11 ± 0.71	3.67 ± 0.47	0.56	6.56	<0.001	0.65
Being the patient’s advocate	3.31 ± 0.64	3.67 ± 0.53	0.36	4.41	<0.001	0.43
Breaking bad news	2.62 ± 0.86	3.51 ± 0.65	0.89	7.97	<0.001	0.79
Unexpected patient deterioration	3.16 ± 0.70	3.71 ± 0.46	0.55	6.53	<0.001	0.64
Care perceived not in patient’s best interest	2.68 ± 0.90	3.56 ± 0.65	0.88	8.11	<0.001	0.80
Communicating within MDT	3.25 ± 0.72	3.72 ± 0.49	0.47	5.42	<0.001	0.53
Dealing with upset/angry relatives	2.99 ± 0.72	3.62 ± 0.53	0.63	6.82	<0.001	0.67
Challenging poor practice	2.98 ± 0.77	3.60 ± 0.65	0.62	6.24	<0.001	0.61
Knowledge tested in clinical environment	3.12 ± 0.63	3.65 ± 0.52	0.53	6.47	<0.001	0.64
Subscale 2 total (12 items)	36.26 ± 7.01	43.56 ± 5.43	7.30	8.30	<0.001	0.82
Overall confidence (21 items)	63.74 ± 11.74	76.51 ± 9.26	12.78	8.83	<0.001	0.87

All items were scored on a 0–4 scale (0 = no confidence, 4 = high confidence). Paired-samples *t*-tests compare pre- and post-intervention scores (*n* = 103). Cohen’s d represents the effect size based on the SD of the difference scores.

### 4.4 Differences across demographic groups

#### 4.4.1 Gender and marital status

Independent-samples *t*-tests were conducted to examine whether confidence differed by gender or marital status. No statistically significant differences were found in either pre-test, post-test, or change scores. Male and female nurses demonstrated comparable gains following the program, as did married and single participants (see Table 4).

**TABLE 4:** Pre–post confidence differences by gender and marital status.

Group	Pre-test M ± SD	Post-test M ± SD	Δ (Post–Pre)	*t*(df)	*p*
Gender					
Male (*n* = 17)	67.6 ± 11.25	75.9 ± 9.22	8.29	–0.45	0.651
Female (*n* = 86)	63.0 ± 11.76	76.6 ± 9.32	13.61		
Marital status					
Single (*n* = 11)	65.3 ± 9.11	77.3 ± 8.08	12.00	–1.48	0.141
Married (*n* = 92)	63.6 ± 12.05	76.4 ± 9.43	12.75		

Negative *t*-values reflect slightly higher mean differences in the comparison group. Analyses used Welch’s correction where variances differed.

The absence of significant gender or marital-status differences indicates that the Charge Nurse Development Program was effective across diverse personal backgrounds. Comparable change scores suggest the curriculum supported learning equally well for all participants, reinforcing the universal relevance and inclusivity of structured leadership development.

#### 4.4.2 Credential, nationality, age, qualification, experience, and unit

Welch’s ANOVA indicated a statistically significant difference in baseline confidence by credential [Welch’s *F*(2, 37.2) = 3.92, *p* = 0.028], with Charge Nurses reporting higher pre-test confidence than Staff Nurses and Team Leaders. However, post-test differences were not statistically significant [Welch’s *F*(2, 38.8) = 1.94, *p* = 0.158], suggesting that all groups converged to similar confidence levels following the intervention. Across nationality, age group, qualification, clinical unit, and years of experience, no significant differences were observed at either pre-test or post-test. Effect sizes were small (ω^2^ < 0.03), indicating minimal practical influence of demographic variables on confidence outcomes.

These findings suggest that although Charge Nurses entered the program with a confidence advantage, the structured intervention appeared to minimize baseline disparities and promote comparable confidence gains across professional and demographic groups. The lack of post-intervention differences supports the potential equalizing role of structured leadership development within a multicultural nursing workforce; however, categories with very small sample sizes (e.g., *n* < 5) should be interpreted cautiously (see Table 5).

**TABLE 5:** Pre- and post-intervention confidence scores by credential, nationality, age, qualification, experience, and clinical unit.

Variable	Group	Pre-test M ± SD	Post-test M ± SD	Test statistic	*P*-value	Effect size
Credential	Staff Nurse (*n* = 58)	61.6 ± 12.05	76.2 ± 8.88	Welch’s *F*(pre) (2, 37.2) = 3.92	0.028	ω^2^ = 0.05
				Welch’s *F*(post) (2, 38.8) = 1.94	0.158	ω^2^ = 0.01
	Team Leader (*n* = 30)	64.1 ± 9.75	75.3 ± 10.58			
	Charge Nurse (*n* = 15)	71.3 ± 11.74	80.1 ± 7.37			
Nationality	Expatriate Asian (*n* = 68)	62.6 ± 11.68	76.0 ± 9.41	Welch’s *F*(pre) (4, 4.92) = 0.73	0.607	ω^2^ = 0.00
				Welch’s *F*(post) (4, 4.88) = 0.48	0.749	ω^2^ = 0.00
	Expatriate Arab (*n* = 23)	64.0 ± 11.94	78.4 ± 7.98			
	Emirati (*n* = 7)	68.6 ± 8.00	79.0 ± 7.57			
	Expatriate African (*n* = 3)	72.0 ± 16.52	69.0 ± 16.09			
	Expatriate Western (*n* = 2)	70.0 ± 18.38	73.5 ± 14.85			
Age group	25–33 years (*n* = 31)	62.8 ± 12.04	77.5 ± 8.38	Welch’s *F*(pre) (3, 10.78) = 2.13	0.155	ω^2^ = 0.03
				Welch’s *F*(post) (3, 4.77) = 0.28	0.840	ω^2^ = 0.00
	34–44 years (*n* = 53)	63.5 ± 11.44	75.7 ± 9.73			
	45–54 years (*n* = 17)	65.7 ± 13.16	77.5 ± 9.37			
	Under 25 years (*n* = 2)	68.5 ± 2.12	73.5 ± 14.85			
Qualification	Diploma (*n* = 6)	58.7 ± 15.06	70.0 ± 8.72	Welch’s *F*(pre) (2, 11.6) = 2.98	0.090	ω^2^ = 0.04
				Welch’s *F*(post) (2, 12.1) = 2.17	0.156	ω^2^ = 0.02
	BSN (*n* = 81)	63.0 ± 11.53	76.5 ± 9.35			
	MSc (*n* = 16)	69.6 ± 10.15	78.8 ± 8.29			
Years of experience	1–5 years (*n* = 8)	65.3 ± 12.12	78.3 ± 8.70	Welch’s *F*(pre) (2, 16.5) = 0.19	0.829	ω^2^ = 0.00
				Welch’s *F*(post) (2, 16.9) = 0.65	0.537	ω^2^ = 0.00
	6–10 years (*n* = 18)	62.4 ± 11.35	78.3 ± 8.76			
	>10 years (*n* = 77)	63.9 ± 11.92	75.9 ± 9.46			
Clinical unit	Medical–surgical inpatients (*n* = 32)	61.5 ± 13.81	79.5 ± 6.90	Welch’s *F*(pre) (7, 24.2) = 1.73	0.149	ω^2^ = 0.03
				Welch’s *F*(post) (7, 22.9) = 1.47	0.226	ω^2^ = 0.02
	Critical care (*n* = 23)	62.3 ± 9.77	76.2 ± 8.91			
	Operating room (*n* = 12)	66.7 ± 10.97	73.3 ± 12.25			
	Outpatient (*n* = 11)	67.2 ± 9.76	79.8 ± 6.34			
	Pediatric Med–Surg (*n* = 7)	62.7 ± 10.67	70.3 ± 10.73			
	Emergency (*n* = 6)	73.2 ± 7.78	73.0 ± 13.48			
	Health support services (*n* = 7)	66.0 ± 13.56	77.0 ± 9.90			
	Maternity (*n* = 5)	57.0 ± 10.70	71.8 ± 8.61			

### 4.5 Reliability of the Confidence Scale

Cronbach’s alpha for the 21-item Confidence in Managing Challenging Situations Scale (CMCS) was 0.963, indicating excellent internal consistency and supporting the tool’s reliability in the UAE context (see Table 6).

**TABLE 6:** Internal consistency analysis of the Confidence Scale.

Items	α if item deleted
1. Understand the legislation relevant to the protection of vulnerable adults	0.9615
2. Support and promote the health, well-being, rights, and dignity of people	0.9610
3. Advocate on behalf of patients and manage ethical challenges	0.9608
4. Work in partnership with service users, carers, and families	0.9612
5. Build partnerships and therapeutic relationships	0.9610
6. Enable patients to make their own decisions and choices	0.9603
7. Recognize distress and respond effectively	0.9603
8. Act as a change agent and provide leadership	0.9605
9. Be self-aware and understand how values and assumptions affect practice	0.9605
10. Manage an unexpected clinical emergency (falls, collapse, seizure)	0.9600
11. Manage a group of patients when mentor is unexpectedly off duty	0.9602
12. Caring for a dying patient	0.9628
13. A cardiac arrest	0.9615
14. Being the patient’s advocate	0.9606
15. Breaking bad news	0.9620
16. Unexpected patient deterioration	0.9600
17. Being asked to undertake care not in the patient’s best interest	0.9644
18. Communicating within the multidisciplinary team	0.9620
19. Dealing with upset or angry relatives	0.9601
20. Challenging poor practice	0.9606
21. Your knowledge being tested within the clinical environment	0.9606

Alpha was calculated for the full 21-item scale. Overall Cronbach’s α = 0.963; item–deleted α-values ranged from 0.960 to 0.964.

The internal consistency analysis demonstrated that the 21-item Confidence in Managing Challenging Situations Scale (CMCS) performed exceptionally well within this sample. Cronbach’s alpha for the overall scale was 0.963, confirming excellent reliability. Item-deleted alpha values were tightly clustered between 0.960 and 0.964, indicating that all items contributed meaningfully to the construct and that no single item reduced the coherence of the scale. This high level of internal consistency suggests strong structural stability and supports the suitability of the instrument for evaluating leadership and clinical confidence among a diverse, multinational nursing workforce. The robustness of the scale strengthens confidence in the validity of the observed improvements following participation in the Charge Nurse Development Program.

### 4.6 Kirkpatrick Level 1 and Level 2 outcomes

The Charge Nurse Development Program was evaluated using the first two levels of the Kirkpatrick Model, a widely recognized framework for measuring training effectiveness in healthcare education.

Kirkpatrick Level 1 (Reaction) captures participants’ satisfaction, perceived relevance, and overall experience of the program. Reaction outcomes were assessed using the post-course evaluation survey completed by all participants. Items included satisfaction with the content, teaching methods, and applicability to clinical practice. Participants reported high levels of satisfaction with all components of the Charge Nurse Development Program. The majority rated the speaker effectiveness as Excellent (82.8%) or Very Good (14.1%), yielding a high-satisfaction rate of 96.9% (*M* = 4.80 ± 0.48). Similar patterns were observed for content quality (96.9% high satisfaction; *M* = 4.75 ± 0.50), audiovisual materials (96.9% high satisfaction; *M* = 4.73 ± 0.52), and the usefulness of facilitated discussions (95.3% high satisfaction; *M* = 4.74 ± 0.55).

Regarding perceived learning value, 40.6% indicated that the educational activity represented “All New” knowledge, 43.8% reported “Moderate” new knowledge, and 15.6% reported “Some” new knowledge (*M* = 2.25 ± 0.74). No inferential statistical tests are required for Level 1 outcomes, as this level focuses on participants’ immediate perceptions rather than changes over time (see Table 7).

**TABLE 7:** Kirkpatrick Level 1 reaction outcomes.

Item	% excellent	% very good	% good	High satisfaction (%)	Mean ± SD
Speaker	82.8%	14.1%	3.1%	96.9%	4.80 ± 0.48
Content	78.1%	18.8%	3.1%	96.9%	4.75 ± 0.50
Audiovisual	76.6%	20.3%	3.1%	96.9%	4.73 ± 0.52
Discussion	78.1%	17.2%	4.7%	95.3%	4.74 ± 0.55
New knowledge	40.6% all new	43.8% moderate	15.6% some	–	2.25 ± 0.74

Kirkpatrick Level 2 (Learning) Kirkpatrick Level 2 evaluates the extent to which participants improved in knowledge, confidence, clinical judgment, and leadership readiness as a result of the Charge Nurse Development Program (see Table 8). In this study, Level 2 learning outcomes were measured using the Phase 2 knowledge test and the pre–post confidence assessment.

**TABLE 8:** Knowledge test pre–post results (Kirkpatrick Level 2: learning).

Variable	Pre-test M ± SD	Post-test M ± SD	Δ Mean	95% CI	*t*(102)	*P*-value	Cohen’s d
Knowledge score	12.07 ± 1.95	13.60 ± 0.70	1.53	[1.20, 1.87]	9.08	<0.001	0.90

Participants demonstrated significant improvement in knowledge following the didactic component of the program. Pre-test scores (*M* = 12.07, SD = 1.95) increased to post-test scores (*M* = 13.60, SD = 0.70), yielding a mean gain of 1.53 points (95% CI [1.20, 1.87]). This improvement was statistically significant, *t*(102) = 9.08, *p* < 0.001, with a large effect size (Cohen’s *d* = 0.90), indicating substantial learning at the cognitive level.

Consistent gains were also observed in leadership and clinical confidence. Subscale 1 (Leadership & Ethical Practice) increased from 27.49 ± 5.31 to 32.95 ± 4.05 (Δ = 5.48), while Subscale 2 (Clinical & Communication Confidence) increased from 36.26 ± 7.01 to 43.56 ± 5.43 (Δ = 7.30). Total confidence improved from 63.75 ± 11.74 to 76.51 ± 9.26 (Δ = 12.78). All pre–post differences were statistically significant (*p* < 0.001), with large effect sizes (*d* = 0.82–0.87 for subscales and total score). These findings provide strong evidence of learning and reinforced competence attributable to the CNDP.

The significant improvements in both knowledge and confidence demonstrate a robust Level 2 learning effect, indicating that the program effectively strengthened participants’ cognitive understanding, clinical judgments, and perceived readiness to lead.

### 4.7 Predictors of change in confidence

A multiple linear regression analysis was conducted to explore whether demographic or professional characteristics predicted changes in total confidence following the CNDP. Predictor variables included age group, gender, nationality group, nursing qualification, years of experience, clinical unit, and credential. The overall model explained 28.5% of the variance in confidence change (R^2^ = 0.285); however, the adjusted R^2^ was low (Adj. R^2^ = 0.078), and the model did not reach statistical significance, *F*(23, 79) = 1.37, *p* = 0.155. Therefore, no robust demographic or professional predictors of confidence change were identified. Given the non-significant overall model and the small size of several subgroups, individual predictor estimates were not interpreted further. These findings suggest that confidence gains were broadly distributed across participants rather than being reliably explained by demographic or professional characteristics (see Table 9).

**TABLE 9:** Overall regression model predicting change in total confidence.

Model	R^2^	Adjusted R^2^	*F*(df)	*P*-value
Demographic and professional predictors of confidence change	0.285	0.078	*F*(23, 79) = 1.37	0.155

## 5 Discussion

This study evaluated the effectiveness of a structured Charge Nurse Development Program (CNDP) in enhancing nurses’ confidence in a large multicultural tertiary hospital in the United Arab Emirates. Significant improvements were observed across all 21 items and both subscales of the Confidence Scale, indicating broad strengthening of leadership, ethical decision-making, clinical judgment, communication, and interpersonal confidence. These gains were consistent across demographic and professional groups, supporting the program’s universal applicability in diverse nursing environments.

The substantial pre–post improvements align with recent international evidence demonstrating the value of targeted leadership development for frontline nurse leaders. Jubinville et al. (15) found that structured leadership workshops enhanced charge nurses’ delegation, communication, and coordination skills closely mirroring improvements seen in this study. Jonson et al. (34) similarly reported that simulation-based leadership training heightened nurses’ self-efficacy in managing unexpected deterioration, consistent with the large gains observed here in emergency response and clinical prioritization. Findings also correspond with Qtait and Jaradat (35) who showed that standardized leadership education reduced variability in leadership behaviors among expatriate nurses in Gulf-region hospitals. This parallels the equalizing effect observed in the present study, where initial credential-based differences disappeared post-intervention.

The absence of significant differences across nationality, age, qualification, clinical unit, and years of experience further demonstrates the CNDP’s adaptability to multicultural nursing settings. This aligns with Bandura’s self-efficacy theory, which emphasizes mastery experiences, vicarious learning, social persuasion, and emotional regulation as foundational mechanisms for developing confidence. The program integrated these elements through simulation, case-based learning, reflective debriefing, and structured feedback, likely contributing to the consistently strong outcomes. The excellent internal consistency of the Confidence Scale (α = 0.963) supports the reliability of these findings, while the large effect sizes (d ≈ 0.8–0.9) indicate that improvements were both statistically and educationally meaningful. Prior work has linked improved leadership confidence to enhanced team climate, reduced adverse events, and increase staff job satisfaction (15), suggesting that the CNDP may contribute to broader organizational benefits.

### 5.1 Implications for nursing leadership, education, and policy

For nursing leadership, the CNDP provides a scalable model for strengthening frontline decision-making, communication, and situational awareness competencies central to charge nurse performance. The universal confidence gains suggest that structured pathways can accelerate leadership readiness among emerging leaders while reinforcing capabilities among experienced staff.

For nursing education, the findings highlight the value of simulation, scenario-based learning, and reflective debriefing in building both technical and interpersonal leadership capacities. Incorporating structured leadership curricula into transition programs, CPD frameworks, and residency models may enhance preparedness for supervisory roles.

For policy and governance, the demonstrated effectiveness of a standardized, culturally informed leadership program supports integrating structured charge nurse preparation into institutional career progression models. Such programs can promote equitable leadership development, strengthen workforce stability, and align with competency-based leadership development particularly in multicultural environments like the UAE.

## 6 Limitations

This study has several limitations. First, the single-group pre–post design without a control group limits the ability to draw firm causal conclusions, as improvements in confidence cannot be definitively attributed to the Charge Nurse Development Program alone. Second, participants were recruited from one tertiary hospital in the UAE using a nomination-based and voluntary participation approach. Although this ensured that the program reached nurses with relevant leadership potential, it may have introduced selection and self-selection bias, as participants may have been more motivated or more ready for leadership development than non-participants. In addition, participant attrition may also have introduced bias, as the characteristics and reasons for non-completion among participants who did not complete all phases and assessments were unavailable for comparison. Third, the study measured self-reported confidence rather than directly observed leadership behaviors or objective clinical, patient, or organizational outcomes. Therefore, while the findings demonstrate meaningful gains in perceived readiness, they do not confirm whether these gains translated into sustained changes in charge nurse practice, team functioning, patient outcomes, or workforce indicators. Finally, the post-assessment was conducted shortly after program completion, which did not allow evaluation of long-term retention or transfer of learning into routine clinical practice. Future research should consider multi-site designs, comparison or control groups, objective behavioral assessments, and longitudinal follow-up to examine whether improvements in confidence are sustained over time and reflected in measurable practice and organizational outcomes.

## 7 Conclusion

This study demonstrated that the CNDP was associated with significant improvements in nurses’ self-reported confidence across professional, ethical, clinical, and communication domains. The findings suggest that structured charge nurse development may support perceived readiness for frontline leadership responsibilities in a multicultural tertiary hospital context. However, because the study used a single-group pre–post design and measured self-reported confidence rather than observed leadership behavior or clinical outcomes, the results should be interpreted as preliminary evidence of confidence development rather than evidence of demonstrated leadership competence or organizational impact. In the UAE context where multiculturalism, high mobility, and rapid system expansion create unique leadership challenges the present findings underscore the necessity of standardized, competency-based leadership preparation. The CNDP offers a model that can be replicated or scaled across institutions seeking to build capacity among frontline nurses.

## Data Availability

The raw data supporting the conclusions of this article will be made available by the authors, without undue reservation.
